# Radiolabelled Peptides for Positron Emission Tomography and Endoradiotherapy in Oncology †

**DOI:** 10.3390/ph13020022

**Published:** 2020-01-30

**Authors:** Christine Rangger, Roland Haubner

**Affiliations:** Department of Nuclear Medicine, Medical University of Innsbruck, Anichstrasse 35, 6020 Innsbruck, Austria; christine.rangger@i-med.ac.at

**Keywords:** radiolabelled peptides, positron emission tomography, peptide receptor radiotherapy, oncology, metabolic stability, labelling strategies

## Abstract

This review deals with the development of peptide-based radiopharmaceuticals for the use with positron emission tomography and peptide receptor radiotherapy. It discusses the pros and cons of this class of radiopharmaceuticals as well as the different labelling strategies, and summarises approaches to optimise metabolic stability. Additionally, it presents different target structures and addresses corresponding tracers, which are already used in clinical routine or are being investigated in clinical trials.

## 1. Introduction

More than two decades ago, radiolabelled (DOTA^0^, Phe^1^, Tyr^3^) octreotide (DOTATOC) was introduced for theranostics of patients with somatostatin expressing neuroendocrine tumours [1]. Only a few years later, the first clinical studies with the ^68^Ga-labelled analogue were carried out [2], paving the way for the development of a variety of different radiolabelled peptides and peptide analogues for the diagnosis and treatment of tumours. In this review, pros and cons of peptides compared to other radiopharmaceuticals like monoclonal antibodies and small molecular weight molecules, labelling strategies using radiohalogens as well as radiometals, and strategies to improve the stability of radiolabelled peptides are discussed. In the second part, the most prominent derivatives used for positron emission tomography (PET) and peptide receptor radiotherapy (PRRT), including peptides targeting somatostatin receptors (SSTR), integrins, chemokine receptors, or the prostate-specific membrane antigen (PSMA), are introduced and their imaging properties are described.

## 2. Peptides as Targeting Probes—Pros and Cons

Radiolabelled small peptides are the major class of radiopharmaceuticals used in the diagnosis and therapy of tumours. This can be explained by the fact that a great diversity of small peptides function as endogenous ligands in a variety of molecular processes during tumour development, growth, signal transduction, and dissemination, as well as that these compounds possess beneficial properties as targeting probes in nuclear medicine.

Compared to antibodies, they are not immunogenic and show fast diffusion and target localisation. Additionally, peptides can be modified, improving metabolic stability and adjusting favourable pharmacokinetics (e.g., see [3]). In contrast to small molecular weight compounds, peptides are more tolerant of modifications, allowing appropriate labelling. For example, chelating moieties for radiometallation are much easier to conjugate to peptides as to small organic structures without interfering with the binding affinity and pharmacokinetic properties. The same applies to modifications to optimise pharmacokinetics like the use of polyethylene glycol (PEGylation), the introduction of carbohydrates or other pharmacokinetic modifiers [3].

Because of the presence of endogenous enzymes for the degradation of peptides and proteins, the major disadvantage compared to small molecular mass probes could be the lower metabolic stability, which is why a variety of strategies exists to overcome this problem. These strategies include the introduction of unnatural amino acids, backbone cyclisation and modifications and are described in detail in the following chapter.

## 3. Improvement of Metabolic Stability

Peptides and proteins are essential endogenous components forming the human proteome. Thus, a variety of enzymes is found in blood and other tissue such as the liver and kidneys to process these compounds. These enzymes include exopeptidases to cleave N- and C-terminal amide bonds as well as endopeptidases, which cleave amide bonds within the molecule sequence.

A variety of strategies, not only in radiopharmaceutical chemistry, focus on the stabilisation of peptides and proteins. Here should be mentioned that protection from exopeptidases might be easier than from endopeptidases. A straightforward strategy for this approach is backbone cyclisation where the N-terminal end is conjugated with the C-terminal end of the peptide sequence. This has been successfully demonstrated with many radiopharmaceuticals targeting integrins. A variety of RGD-peptides—binding to integrin receptors—are based on the cyclic pentapeptide c(RGDfV), which shows high stability in vivo (for a review, see, e.g., [4]). For example, no metabolites of [^68^Ga]Ga-NODAGA-RGD (where NODAGA is 1,4,7-triazacyclononane,1-glutaric acid-4,7-acetic acid) were found in the blood and urine of patients over the whole observation period up to 1 h post-injection [5]. However, problematic might be that, due to cyclisation, the flexibility of the peptide is reduced which, in the case of the RGD-peptides, improved the target selectivity but can also negatively influence the affinity of the peptide. Thus, this approach cannot be seen as a general strategy to improve the metabolic stability of peptides. In some cases, modification of the N- and/or C-terminal end also allows protection from exopeptidases. Especially, if radiometallation for labelling is used, corresponding chelating moieties have to be introduced. In many cases, these systems are conjugated either directly or via a spacer to the N-terminal end, which might already increase stability against exopeptidases. The C-terminal modifications often include the formation of an amide (-CONH_2_ or -CONHR) as found for, e.g., radiolabelled minigastrin and bombesin derivatives [6,7]. Reduction of the carboxylate to an alcohol function is applied with Tyr^3^-octreotide, where the C-terminus includes a threoninol instead of a threonine [8].

Another relatively easy strategy for stability improvement towards endopeptidases is based on the use of d-amino acids or unnatural amino acids such as naphthylalanine, phenylglycine, norleucine, and cyclohexylalanine as found, e.g., by Murza et al. [9] or Klingler et al. [10]. In many cases, a combination of N/C-terminal modification with stabilisation via d-amino acids or unnatural amino acids is used as described for Tyr^3^-octreotide or different RGD-derivatives. The sequence of Tyr^3^-octreotide includes N-terminal d-Phe and d-Trp at position 4 in combination with the C-terminal threoninol, as already referred to above. For a variety of RGD-based tracers like Galacto-RGD, backbone cyclisation is combined with the introduction of d-Phe in position 4 of the peptide sequence [11], which, besides stabilisation, also results in the RGD-sequence being in the γ-turn position which is necessary for high integrin α_v_β_3_ affinity [12]. Some endopeptidases recognise special amino acids, e.g., trypsin is specific for Arg and Lys and chymotrypsin for Trp, Tyr and Phe. Thus, the replacement of these amino acids in the corresponding sequence will be of particular benefit. Obviously, not all amino acids in a sequence can be replaced either by the corresponding d-amino acid or by an unnatural amino acid without influencing the binding affinity. Sometimes the essential amino acids of a peptide sequence are already known; if not, a so-called “alanine scan” (for details see, e.g., [13]) may supply this information.

Other stabilisation approaches focus on the modification of amide bonds, which are the cleavage site of endopeptidases. These modifications range from methylation/alkylation of the nitrogen to complete replacement of this moiety, resulting in a variety of peptide bond isosteres [14]. Usually, such modifications are chemically much more complex than the introduction of unnatural amino acids and can result in drastic changes to the peptide properties. Depending on the modification carried out, it includes higher flexibility of the modified amide bond and changes in electron densities and thus, changes in the hydrogen bridge donor/acceptor character. This might alter the peptide structure and could influence the binding affinity as well as the pharmacokinetics. The formation of such peptide bond isosteres is often starting with the synthesis of the corresponding pseudo dipeptide, which will be incorporated in a second step into the peptide sequence. Especially the synthesis of such dipeptide derivatives including complex (e.g., side chain functionality containing) amino acids can result in difficult multistep synthesis routes and determination of the binding affinity of the final peptide after modification is a prerequisite. Nevertheless, the introduction of such peptide bond isosteres has already been successfully applied to stabilise radiolabelled neurotensin analogues [15].

## 4. Labelling Strategies

Two general labelling strategies for peptides can be distinguished: Labelling via radioactive halogen isotopes like fluorine-18, bromine-76, iodine-124, iodine-131, and astatine-211 and labelling via radiometals like copper-64, gallium-68, zirconium-89, yttrium-90, technetium-99m, indium-111, and lutetium-177 (for physical characteristics of the different isotopes, see Table 1). Due to the different characteristics, labelling of the first class focuses on direct labelling or labelling via prosthetic groups, whereas labelling of the latter is carried out via chelating systems coupled with the peptides. Also, most recently, strategies to use fluorine-18 with chelating systems have been introduced. Here we want to summarise some standard labelling protocols, describe the most common prosthetic groups as well as new alternative labelling strategies. With the exception of radioiodine, only labelling procedures for isotopes used with PET or therapeutic approaches will be included.

### 4.1. Halogens

#### 4.1.1. Fluorine

Flourine-18 is the most common radioisotope for PET. It combines with 109 min, an appropriate half-life with high positron emission (97%), and suitable positron energy (0.634 MeV) and can be produced easily from enriched ^18^O-water with every routine cyclotron.

For small molecular mass compounds like [^18^F]FDG, a frequently applied strategy is direct labelling via nucleophilic substitution. This approach is, in many cases, not suitable for peptides due to the harsh reaction conditions and potential formation of [^18^F]HF in the presence of acidic protons. Therefore, although in some cases direct labelling can be applied (see, e.g., [16]), a general strategy for ^18^F-labelling of peptides is based on prosthetic group labelling and uses the different functional groups of the corresponding amino acids like thiols, carboxylates, alcohols, and amines in the peptide sequence. Thus, ^18^F-labelling strategies focusing on alkylation, acylation, and amidation are described [17,18].

Acylation of an amino function in the peptide sequence (N-terminal or ε-amino function of lysine) is a widespread approach of introducing an activated ester as a prosthetic group. The most prominent derivative is *N*-succinimidyl-4-[^18^F]fluorobenzoate ([^18^F]SFB). Based on the three-step synthesis described by Vaidyanathan and Zalutsky [19], a variety of modifications has been suggested to optimise synthesis and radiopharmaceutical yield [20]). For example, Wüst et al. [21] started with the fluorination of 4-*N*,*N*,*N*-trimethylammonium ethyl benzoate followed by the hydrolysis of the ethyl ester and subsequent activation using *O*-(*N*-succinimidyl)-*N*,*N*,*N*’,*N*’-tetramethyluronium tetrafluoroborate (TSTU). Another prosthetic group for labelling peptides is 4-nitrophenyl-2-[^18^F]fluoropropionate [11], which is, due to its aliphatic character, less lipophilic than the benzoic acid derivative. As for a variety of prosthetic groups using acylation for conjugation with the peptide, again, the synthesis includes labelling of the corresponding precursor, hydrolysis of the ester and subsequent activation for acylation. In general, this strategy needs at least one HPLC separation step of the final product; sometimes even intermediates have to be isolated via HPLC, which makes these approaches very complex and time consuming.

The major obstacle in developing an easy 1-step procedure is found in the reactivity of the activated ester function, which interferes with the fluorination reaction [20]. Regardless of that, some 1-step procedures have been reported, including the synthesis of *N*-succinimidyl-4-([^18^F]fluoromethyl)benzoate [22] and [^18^F]fluoronicotinic acid 2,3,5,6-tetrafluorophenyl ([^18^F]Fpy-TFP), a nicotinic acid derived active ester [23]. For the latter, labelling yields have been between 60 and 70% at low temperatures with almost quantitative conjugation, making this approach an interesting alternative for a variety of peptides. Other strategies to obtain 1-step procedures use the high affinity of fluorine to boron and silicone and introduce silicone fluoride acceptor (SiFA) groups [24] for labelling the prosthetic group. Relying on an isotopic exchange, labelling is straightforward. However, the disadvantage is the high lipophilic property of the SiFA group, which can have a negative influence on the pharmacokinetics of especially small peptides.

In the last decades, other options for ^18^F-labelling of peptides have been reported. These consist of conjugation via “click chemistry”, oxime and hydrazone formation, or the use of maleimide as the thiol reactive group (Michael addition) (for a review, see [20]). Click chemistry, the 1,3-dipolar cycloaddition of an alkyne and an azide at ambient temperature, was first used in radiochemistry to produce ^99m^Tc(CO)_3_-labelled radiopharmaceuticals [25]. Recently, this approach was also introduced for ^18^F-labelling. ^18^F-labelled alkynes [26] as well as azides [27] have been reported as prosthetic groups for the labelling of correspondingly modified peptides (e.g., via 3-azido propionic acid or propargylglycine) and demonstrated the fast and regioselective Cu-catalysed formation of the corresponding triazole. More recent applications avoid the use of Cu(I) catalyses and are focused on strain-promoted Cu-free click chemistry with aza-benzocyclooctyne derivatives as constrained dipolarophiles [28,29]. Nonetheless, the elimination of the Cu(I) is paid by an increased lipophilic character of the resulting radiopharmaceutical, which may negatively influence the pharmacokinetics.

Another approach introduces aminooxy-functionalised peptides allowing regioselective labelling using aldehydes and ketones as prosthetic groups. The most commonly used ^18^F-labelled aldehyde is 4-[^18^F]fluorobenzaldehyde [30]. This strategy has also been used to label peptides: for example, Poethko et al. [31] successfully labelled multimeric RGD-peptides modified with an aminooxy function. Interestingly, also 2-hydrazinonicotinic acid (HYNIC)-modified peptides allow reactions with 4-[^18^F]fluorobenzaldehyde via the formation of a hydrazone, which has been demonstrated with HYNIC-Tyr^3^, Thr^8^-octreotide [32]. Radiolabelled carbohydrates could be also an interesting prosthetic group because they would not only allow ^18^F-labelling of the peptide but also increase the hydrophilic character, which is often a prerequisite for favourable pharmacokinetics. In this regard, it has been proven that [^18^F]FDG (which is in the open form an aldehyde) can be conjugated to peptides via oxim formation. Critical is that the routinely produced [^18^F]FDG includes high amounts of free glucose interfering with the labelling reaction, thus a HPLC separation has to be introduced to get acceptable labelling yields [33,34]. Other approaches introduced modified carbohydrates like thiosulfonate derivatives, allowing formation of disulfide bridges with Cys in the peptide sequence [35], or an azide derivatised FDG, which can be conjugated via click chemistry [36], as discussed earlier. 

The chemoselectivity of thiol groups, as found in the amino acid Cys, is also exploited using maleimide derivatives as prosthetic groups (for a review, see [20]). The syntheses of the described derivatives are very complex and include, commonly, 3 to 4 steps [37], or start with another prosthetic group (e.g., 4-[^18^F]fluorobenzaldehyde or [^18^F]SFB) [38,39], which is bound in a subsequent step to a variety of maleimide derivatives. It is questionable if the advantage of the high regioselectivity compensates the high complexity of the synthesis route. Strategies that are more straightforward include isotopic exchange reactions with boron- and silicone-containing maleimide derivatives [40,41] or conjugate the maleimide group to the peptide and use thiol-containing prosthetic groups [42,43].

An alternative strategy, which allows direct labelling of peptides and proteins, is based on the introduction of aluminium fluoride species “AlF” (for an overview, see [44]). This approach uses the strong binding of fluorine to aluminium and allows labelling via chelating systems, according to the labelling with radiometals. Several chelating moieties have been studied, of whom NOTA derivatives might be the best choice, yet [44]. A variety of peptide structures have been labelled using this method. Labelling is straightforward with reaction times of approximately 15 min at 100 °C (see, e.g., [45]). Critical might be the stability of the complexes. However, initial clinical data with RGD-peptides [46,47] and octreotide derivatives [48] demonstrated good delineation of the tumours with low activity in the bone (see also below).

Due to the mild reaction conditions of the SiFA approach, this strategy was not only used for the labelling of prosthetic groups but also for direct labelling of peptides modified with corresponding SiFA groups [49]. To improve the hydrophilic character of such modified peptides, hydrophilic modifiers and the SiFA*lin* building block have been introduced. The resulting ^18^F-SiFA*lin*-Glc-Asp_2_-PEG_1_-TATE could be produced in approximately 10 min at ambient temperature in good radiochemical yield. 

#### 4.1.2. Bromine

Bromine-76 is a positron emitter (54% positron emission) with a half-life of 16.2 h, which is comparably long related to the routinely-used PET isotopes, enabling the use in tracers with a slower elimination from the body. Labelling can be done in analogy to iodine using the electrophilic substitution approach and tyrosine in the peptide sequence (see also below). Oxidation can be carried out via oxidising reagents like chloramine T [50] or via corresponding enzymes [51]. In addition, prosthetic group labelling strategies can be used, including Bolton Hunter labelling [52] or approaches with oxidative substitution and trialkyl tin leaving groups, as found in *N*-succinimidyl p-(4-tri-n-butylstannyl)benzoate [53]. Recently, an electron rich 2,6-dimethoxybenzene moiety was introduced, which can easily attach bromine-76 at its activated position ortho to the methoxy group without the need of a leaving group. Thus, *N*-succinimidyl 2,6-dimethoxybenzoate can be either used for direct labelling through pre-conjugation or as a ^76^Br-labelled prosthetic group for indirect labelling, which has been successfully demonstrated by conjugation to the amino function in the cyclic peptide c(RGDyK) [54].

The main advantage of bromine-76 might be the suitable long half-life, making it an interesting alternative for some applications, but a problem is that a potential radiocatabolite might be radiobromide, which is very slowly excreted, and is distributed in the extracellular space, resulting in high background activity. Thus, careful tracer design and optimisation is required to avoid this obstacle in the introduction of bromine-76 for PET applications [55].

#### 4.1.3. Iodine

A variety of radioactive iodine isotopes is available. These range from iodine-125 for preclinical research and RIA applications over iodine-123 for single-photon emission tomography (SPECT) to iodine-124 for PET and iodine-131 for therapeutic approaches. Labelling strategies are equivalent for all isotopes (for a review, see, e.g., [56]). The easiest way to label peptides is based on the electrophilic substitution of the activated protons in position 2 and 5 of the phenol ring of tyrosine moieties in the peptide sequence. Hence, the iodide in sodium iodide is oxidised to the iodine cation (I^+^). For oxidation, different strategies are available. Typical oxidation reagents are chloramine T, chloramine T bound to a resin (Iodobeads^®^) or Iodogen^®^. The latter two methods have the advantage that, under standard labelling conditions, the separation of the insoluble oxidation reagent is straightforward. Problems can arise if oxidation sensitive amino acids like Cys and Met or His, which can also be iodinated at the ring system, are included in the peptide sequence. In such cases, different options are available: (A) Separation of the I^+^-species from the oxidising reagent before incubation with the peptide (which might result in low labelling yields). (B) The use of peroxidases for the oxidation, which are milder oxidation reagents (but might have the disadvantage that the enzyme itself will be labelled which is why a subsequent separation of the protein from the desired radiolabelled peptide is needed). (C) The use of prosthetic group labelling. From the presented options, (A) and (B) are only of advantage for Cys and Met. The latter strategy can be used in any case and is the approach of choice if no tyrosine is present in the peptide sequence of interest.

The most common prosthetic group for iodination is the Bolton Hunter reagent [*N*-succinimidyl-3-(4-hydroxyphenyl) propionate] [57]. This activated ester is commercially available in the iodinated form, at least for iodine-125. Regardless, the Bolton Hunter reagent can be easily iodinated in-house using the same procedures described for the labelling of tyrosine. Due to the activated carboxylate, the iodinated Bolton-Hunter can be conjugated to any amino function in the peptide sequence—N-terminally as well as on the ε-amino function of lysine. If a regioselective position is desired, potential additional amino functions have to be protected. In many cases, labelling via the electrophilic substitution results in good labelling yields but even better labelling yields might be provided by using *N*-succinimidyl-3-(tri-*n*-butylstannyl)benzoate as the prosthetic group [58]. Compared with the phenol derivative, an additional advantage is the higher metabolic stability of the resulting C-I bond of the benzoate derivative because the latter can be de-iodinated by endogenous enzymes involved in the metabolism of thyroid hormones.

#### 4.1.4. Astatine

Astatine-211 (half-life 7.2 h) is an α-particle emitting radiohalogen (range approx. 25–100 µm; energy approx. 4–8.5 MeV), which was already suggested for targeted radiotherapy of small tumour clusters or even isolated disseminated tumour cells more than 40 years ago [59]. Nonetheless, initial clinical trials with a ^211^At-labelled chimeric monoclonal antibody (mAb) were not described before this millennium [60]. In this case, prosthetic group labelling was carried out using *N*-succinimidyl-3-(tri-*n*-butylstannyl)benzoate as precursor [61]. Since this clinical study, not many more reports were published. In 2009, one additional study reports on [^211^At]At-MX35 F(ab’)_2_ for the treatment of ovarian cancer with a long-term follow-up presented most recently [62,63]. In contrast to Zalutsky et al., another group reported on ^211^At-labelling via a direct procedure on an ε-lysyl-3-(trimethylstannyl)benzamide modified immunoconjugate [64]. 

Theoretically, a variety of labelling strategies might be possible because of the ambivalent character of astatine—being either halogen or metalloid. These involve halogen exchange, dediazoniation, electrophilic aromatic substitution, and demetallation on one hand, and complex formation on the other [65]. Due to the soft-cation character of astatine, chelators with soft-donor atoms like sulphur are superior to chelators with hard-donor atoms, as found in diethylenetriaminepentaacetic acid (DTPA) or ethylenediaminetetraacetic acid (EDTA). This could be demonstrated by comparing the complex formation constants of a variety of chelators [65,66]. Regardless of that, in vivo stability of such complexes seems low, as demonstrated in studies with ^211^At-labelled sulphur-containing calix[4]arene [67]. Thus, most efforts are focused on the development of labelling strategies based on the formation of carbon/astatine bonds [65]. Among the listed approaches, demetallation reactions seem the most appropriate for ^211^At-labelling of peptides and proteins. As already discussed above, tributyl- and trimethylstannyl groups are preferably used as leaving groups. In most cases, labelling via prosthetic groups, especially *N*-succinimidyl astatobenzoate (SAB), is carried out whereas direct labelling was also described. Due to its high energy, the LD_10_ of [^211^At]astatide is low [68] and great emphasis has to be laid on the in vivo stability of corresponding radiopharmaceuticals. Most recently, a [^211^At]At-astatobenzamido-labelled PSMA derivative demonstrating specific prostate cancer cell kill in vitro and in vivo has been introduced [69]. Again, some dehalogenation was observed but the major concern was the late nephrotoxicity due to α-particle irradiation of the tracer bound to receptors in the kidneys. 

Boron clusters have been considered for labelling with astatine-211 because the boron/astatine bond is more stable than the carbon/astatine bond. Fab fragments modified with a variety of boron clusters (e.g., nido-carboranes and closo-decaborates) allowed ^211^At-labelling in yields up to 75% within 10 min and ^211^At-labelled Fab fragments revealed high in vivo stability [70]. However, the introduced boron cluster negatively influenced the pharmacokinetic of the Fab fragment with increased activity found in some tissue, including the liver. 

### 4.2. Radiometals

#### 4.2.1. Copper

The decay characteristics of copper-64 [half-life: 12.7 h; β^+^: 0.653 MeV (17.8%); β^−^: 0.579 MeV (38.4%)] allow PET imaging as well as targeted radiotherapy. Moreover, the long half-life enables the use of peptides and especially proteins with longer blood circulation times in combination with PET. 

Copper exists in several oxidation states, and for radiopharmaceutical approaches, copper (II) possesses the optimal characteristics, making copper (II) complexes the most attractive area for research in clinical radiopharmacy. Copper (II) is a d^9^ metal and coordinates preferably with amines, imines, and bidentate ligands such as bipyridine [71]. Due to the intensive use with other metals like gallium-68, lutetium-177, or yttrium-90, 1,4,7,10-tetraazacyclododecane-1,4,7,10-tetraacetic acid (DOTA) was also studied as a bifunctional chelator for labelling with copper-64. Nonetheless, the in vivo stability of such complexes is suboptimal with transmetallation to endogenous proteins (see, e.g., [72,73]). Slightly higher stability against transchelation is found if 1,4,8,11-tetraazacyclotetradecane-1,4,8,11-tetraaceticacid (TETA) is used for complexation, as shown with [^64^Cu]Cu-TETA-OC [74]. Compared to DOTA and TETA, additional cyclen and cyclam derivatives revealed improved stability in vivo (for an overview, see [75]). Recently, 1,4,7-triazacyclononane-*N,N’,N’’*-triacetic acid (NOTA) derivatives were investigated for the complexation of copper-64 and in several studies, this chelating system demonstrated good performance with high labelling yields and improved stability in vivo [76,77,78].

Another way to improve metabolic stability is focused on cross-bridged macrocyclic chelating agents. The most prominent might be 4,11-bis(carboxymethyl)-1,4,8,11-tetraazabicyclo[6.6.2]hexadecane (CB-TE2A) [79]. The major disadvantage of this chelator is the harsh labelling conditions, which makes it only suitable for heat-insensitive peptides [75]. There are a variety of optimisations for the labelling conditions including the introduction of a propylen instead of the ethylene cross-bridge (PCB-TE2A; [75]) or the use of phosphonate instead of the carboxylate groups (CB-TE2P; [80]). Especially with the latter, labelling can be carried out at room temperature. Most recently, Dos Santos et al. [81] studied different cross-bridged and non-bridged cyclam derivatives conjugated to PSMA for the use with copper-64 and found the best performance of a non-bridged derivative. It was argued that kinetic inertness against demetallation might be more crucial than thermodynamic inertness in vivo and that the overall configuration of the Cu(II)-complex has to be considered.

In addition, sarcophagine-based chelators (hexaazamacrobicyclic cage type ligands like SarAr, AmBaSar, diamSar, and BaBaSar) are studied as bifunctional chelators for labelling with copper-64 [75,82]. These nitrogen-rich chelators allow labelling within a few minutes at room temperature and the resulting complexes show excellent in vivo stability as well as strong resistance to dissociation in vitro (for details, see [82]). A disadvantage might be the lipohilic character, which leads to the formation of cationic or natural Cu-complexes. Thus, derivatives including carboxylate functions like BaBaSar have been introduced. Recently, [^64^Cu]Cu-BaBaSar-RGD2 was studied in non-human primates, demonstrating high metabolic stability and predominantly renal elimination with highest activity concentration in kidneys and urinary bladder wall [83].

#### 4.2.2. Gallium

Since ^68^Ge/^68^Ga-generators became available, there has been a great interest in the development of ^68^Ga-labelled peptides for PET imaging. Besides the broad availability (independent of a cyclotron in-house or nearby), also the straightforward methods for the labelling of peptides and the successful introduction of [^68^Ga]Ga-DOTATOC for imaging SSTR expression in clinical routine were the basis for this widespread interest. Meanwhile, the daily demand of corresponding radiopharmaceuticals increased so that, in some cases, the supply via generator hampers due to the limited activity that can be eluted. Thus, there are great attempts to establish cyclotron-based production routes (for an overview, see [84]) which might overcome this problem, at least where a cyclotron is available.

In general, two types of bifunctional chelator can be distinguished for complexation of gallium-68: acyclic and cyclic derivatives. Acyclic chelators include DTPA, desferrioxamine B (DFO), H_2_dedpa, 1,2-dimethyl-3,4-hydroxypyridinone (deferiprone), and hydroxybenzyl ethylenediamine (HBED) (for more details, see, e.g., [82]). Due to the use with a PSMA targeting radiopharmaceuticals, meanwhile, *N*,*N*′-bis[2-hydroxy-5-(carboxyethyl)benzyl]ethylenediamine-*N*,*N*′-diacetic acid (HBED-CC) might have become the most well-known acyclic derivative for labelling with gallium-68 [85]. More commonly used are macrocyclic derivatives. Initially, DOTA was the chelating moiety of choice but the atom radius of gallium-68 is not optimal for the complexation, which leads to a hexa-coordinated complex not including all carboxylate functions. The nine membered ring structure of NOTA and its derivatives is better suited for gallium-68, resulting in higher complex stability and already allows labelling at room temperature. This could be proven, e.g., with [^68^Ga]Ga-NODAGA-RGD, which was more stable in vivo than [^68^Ga]Ga-DOTA-RGD [86]. Triazacyclononane-phosphinic acid (TRAP) is also a nine membered ring but includes phosphinic acid moieties. The complex-forming constant is high, allowing labelling at room temperature with low amounts of labelling precursor [87]. Due to the presentation of three terminal carboxylate functions, it is possible to use this chelator as a scaffold for multimeric tracers. Notni et al. [88] presented a ^68^Ga-labelled trimeric RGD-containing tracer with good imaging performance. If only monomeric derivatives are of interest, 1,4,7-triazacyclononane-1,4-bis[methylene(hydroxymethyl)-phosphinic acid]-7-[methylene(2-carboxyethyl) phosphinic acid] (NOPO) is an alternative to TRAP where the phosphor group of two arms is oxidised to phosphonic acid [89]. Another class of bifunctional chelator is 1,4-bis(carboxymethyl)-6-[bis(carboxymethyl)]amino-6-methylperhydro-1,4-diazepine (AAZTA) with a N_3_O_3_ complex geometry. In contrast to the before-mentioned nine membered ring systems, they combine a macrocyclic 1,4-diazapam ring with a free acyclic arm [90]. Recently, also siderophores, cyclic DFO derivatives used especially by fungi for iron complexation, have been introduced for complexation of gallium-68 [91,92]. Depending on the derivative used in analogy to the TRAP/NOPO system, monomeric as well as multimeric radiopharmaceuticals can be produced.

#### 4.2.3. Zirconium

Zirconium-89 is a transition metal of group IVB. The decay proceeds via electron capture (77%) and positron emission (23%) with an Emax of the positron decay of 902 keV and a half-life of 3.3 days. The comparable long half-life makes it an ideal isotope for the use with mAbs and mAb fragments. Thus, developments are primarily focused on immuno-PET applications introducing a variety of ^89^Zr-labelled antibodies including [^89^Zr]Zr-trastuzumab (anti-HER2 mAb) [93], [^89^Zr]Zr-cetuximab (block EGFR activation) [94], and [^89^Zr]Zr-bevacizumab (block VEGF-induced tumour angiogenesis) [95]. Of disadvantage might be the high gamma emission at 909 keV, which limits the radioactive dose that can be administered.

The most commonly used chelating systems are based on DFO, which conjugates Zr^4+^ via the three hydroxamate moieties of the compound [96]. Since Zr^4+^ is preferable, octa-coordinated DFO seems not to be the optimal chelating system. This instability could be shown in in vivo studies where some release of the metal with up to 10% ID/g of the radionuclide was found in the bone [97]. Despite this disadvantage, a variety of bifunctional chelators based on DFO have been developed. They can be divided into two groups: derivatives binding randomly preferred on amino functions of lysine moieties in the sequence or derivatives binding side specifically via, e.g., thiol-reactive groups or click chemistry (for a review, see [97]).

Improvement of complex stability is also achieved by adding an additional hydroxamate group [98]. The resulting DFO*-pPhe-NCS-conjugated [^89^Zr]Zr-trastuzumab showed reduced bone, spleen, and liver uptake in tumour-bearing nude mice compared to the [^89^Zr]Zr-trastuzumab conjugated with commercially available DFO [99]. It is known that a macrocyclic system improves complex stability; thus, some attempts are also made to develop such structures for the complexation of zirconium-89. These strategies include the introduction of natural siderophores like fusarinine C (FSC) and their derivatives [100], cyclic compounds containing 1-hydroxypyridin-2-one groups (HOPO) [101], and DOTA [102]. The use of DOTA for Zr^4+^ is especially surprising because this system offers only four oxo-coordination sites; the remaining coordination sites are covered by nitrogen atoms. Despite that, the resulting complex shows high in vitro and in vivo stability. Of disadvantage are the drastic labelling conditions (95 °C, 1 h) which make the use with antibodies problematic. To overcome this problem, it is discussed to use DOTA-derivatives as prosthetic groups and label the antibody before conjugation to the biomolecule [97].

Altogether, there are many alternative approaches studied, but all of them have some disadvantages; thus, further developments are needed to find the optimal bifunctional chelator for zirconium-89. However, in most clinical settings, mAbs modified with DFO are still used because this bifunctional chelator is readily available, and the eventual release of zirconium from the chelator seems not to influence imaging quality.

#### 4.2.4. Yttrium, Lutetium

Yttrium-90 (half-life 2.7 days) and lutetium-177 (half-life 6.7 days) are both β^-^-emitting isotopes used for endoradiotherapy. In contrast to yttrium-90, lutetium-177 also emits γ-radiation, and its distribution can be monitored using SPECT. For lutetium-177, Eβ_max_ is 0.5 MeV and 2.3 MeV for yttrium-90, resulting in a maximum tissue penetration range of 1.5 and 12 mm, respectively [103].

Coordination chemistry and properties of Y^3+^ and Lu^3+^ are comparable. Both prefer 8 to 9 hard ligand donor atoms like carboxylate oxygen or amine nitrogen for coordination, resulting in antiprismatic or monocapped square antiprismatic geometries [82]. The current standard chelator for complexing these isotopes is DOTA. An accepted disadvantage of DOTA-labelling is that, due to the slow labelling kinetics, sufficient labelling yields are only achieved with enhanced temperatures [82]. An alternative for DOTA would be 2-(p-isothiocyanatobenzyl)-cyclohexyldiethylenetriamine pentaacetic acid (CHX-A′′-DTPA), which showed enhanced radiolabelling kinetics [82]. Nonetheless, labelling with yttrium-90 or lutetium-177 often requires mild heating (>37 °C) and reaction times between 30 and 60 min to achieve reasonable labelling yields.

Attempts have been made to develop chelator systems allowing labelling under so-called “kit conditions” (room temperature, pH 5–6). Recently, 6-Amino-6-(5-methoxy-5-oxopentyl)-1,4-diacepine-tetraacetate (AAZTA-5) was introduced and demonstrated that by conjugation to TOC labelling with lutetium-177 in high yield was possible with low tracer amounts in less than 10 min at room temperature at a pH between 4.5 and 5.5 [104]. Stability in human serum was acceptable even after 7 days of incubation. Another study investigated *N*,*N*′-bis(6-carboxy-2-pyridylmethyl)ethylenediamine-*N*,*N*′-diacetic acid (H_4_octapa) as an acyclic chelator exhibiting significant thermodynamic stability and kinetic inertness with lutetium-177 [105]. Moreover, H_4_octapa-trastuzumab could be labelled with lutetium-177 at room temperature within 15 min in high radiochemical yield and was found to be superior to DOTA-trastuzumab. It is assumed that this chelator shows comparable properties if labelled with yttrium-90, but this is not yet proven. Another macrocyclic chelator system discussed for labelling with yttrium-90 and lutetium-177 is {4-[2-(Bis-carboxy-methylamino)-5-(4-isothiocyanatophenyl) pentyl]-7-carboxymethyl[1,4,7] triazonan-1-yl}acetic acid (3p-C-NETA-NCS) [106]. Again, 3p-C-NETA-trastuzumab can be labelled at room temperature in short reaction times in high radiochemical yield but, in contrast to the before-mentioned, this works for both lutetium-177 and yttrium-90.

Despite these promising data, none of the alternative bifunctional chelators are applied in ^90^Y- or ^177^Lu-labelled radiopharmaceuticals in clinical routine, yet this might change if more sensitive mAb or other proteins become interesting for endoradiotherapeutic applications.

#### 4.2.5. Actinium

Actinium-225 (half-life 10.0 d) decays by emission of net 4 α- (energy between 5.8 and 8.4 MeV) and 2 β^−^-particles (energy between 198 and 659 keV) to bismuth-209 and is, thus, recognised as a potential isotope for the endoradiotherapy of cancer.

Actinium isotopes are typically found as 3+ cations. Due to the large ionic radius (112 pm) large polydentate chelators should be the compounds of choice [107]. As for other radioisotopes, macrocyclic chelators seem to be superior. There is a variety of chelators studied for complexation of ^225^Ac^3+^. These include 1,4,7,10,13,16-hexaazacyclohexadecane-*N*,*N*′,*N*″,*N*‴,*N*′‴,*N*″‴-hexaacetic acid (HEHA) [108] and DOTA as well [109]. However, HEHA-mAb-constructs did not internalise and released actinium-225 from the complex, resulting in liver and bone accumulation [110]. In contrast, [^225^Ac]Ac-DOTA-mAb-conjugates were more stable in vivo and formed internalising immune complexes with the corresponding antigen [110]. As already mentioned, a disadvantage is the elevated temperature needed for high labelling yields, which limits the application of this chelator for sensitive biomolecules such as antibodies [107]. Again, the use of [^225^Ac]Ac-DOTA-derivatives as prosthetic groups is suggested to solve this problem [111]. Recently, *N*,*N*’-bis[(6-carboxy-2-pyridil)methyl]-4,13-diaza-18-crown-6 (H_2_macropa) and its bifunctional analogue H_2_macropa-NCS have been proposed [112]. This chelator allows labelling with actinium-225 at room temperature within several minutes at submicromolar precursor concentrations. ^225^Ac-labelled trastuzumab as well as [^225^Ac]Ac-macropa-RPS-070 (targeting PSMA) showed high in vivo stability and good tumour targeting properties with low activity found in other organs, making it an interesting alternative in complexing actinium-225.

A summary of the different chelators which are used for labelling with radiometals and the corresponding labelling conditions are presented in Table 2.

## 5. Clinically Investigated or Routinely Used Radiolabelled Peptides

A great variety of peptides have been explored for use as radiolabelled probes for tumour diagnosis and treatment (see, e.g., [123,124]). Examples are peptides targeting SSTR, PSMA, integrins, chemokines, urokinase-type plasminogen activator receptors (uPAR), cholecystokinin receptors (CCK2-R), and bombesin receptors. Due to the enormous amount of compounds described, we will focus on target structures where derivatives are already in clinical routine or at least in clinical studies.

### 5.1. Somatostatin Receptor

Radiolabelled octreotide derivatives are the most well-known and best-established peptide-based radiopharmaceuticals for diagnosis as well as treatment of neuroendocrine tumours (NET) (for an overview, see, e.g., [125]). One of the frequently used tracers for PET is [^68^Ga]Ga-DOTATOC [126], which combines easy accessibility with good targeting properties. Labelling with gallium-68 is possible without an in-house cyclotron using corresponding generators but also limits the daily amount of possible investigations. [^68^Ga]Ga-DOTATOC mainly targets SSTR-2 and, to a lesser extent, SSTR-5. Other radiolabelled PET tracers with comparable clinical accuracy are [^68^Ga]Ga-DOTA-Tyr^3^-octreotate ([^68^Ga]Ga-DOTATATE; mainly SSTR-2 targeting) and [^68^Ga]Ga-DOTA-1-Nal^3^-octreotide ([^68^Ga]Ga-DOTANOC; targeting SSTR-2 and SSTR-5 and, to a lesser extent, also SSTR-3,) [8,127,128,129].

At present, only ^68^Ga-labelled octreotide derivatives are used in clinical routine with PET but there are approaches to establish also ^64^Cu- and ^18^F-labelled analogues [130]. Already in 2001, a first in human study with [^64^Cu]Cu-TETA-octreotide was described [131]. In this trial with two patients, [^64^Cu]Cu-TETA-octreotide demonstrated superior imaging properties compared to OctreoScan^®^ but also showed low in vivo stability. Pfeifer et al. evaluated [^64^Cu]Cu-DOTATATE in a first in human study in 14 NET patients [132]. The images revealed high spatial resolution with good tumour-to-background ratios on early as well as late PET scans. Despite some release of copper-64, in vivo stability of the tracer seemed to be sufficient for imaging purposes. A comparison of [^64^Cu]Cu-DOTATATE and [^68^Ga]Ga-DOTATOC demonstrated that both compounds perform equally but [^64^Cu]Cu-DOTATATE detected significantly more additional true-positive lesions, which was explained by the shorter positron range of copper-64 [133]. Wester et al. [134] developed an ^18^F-labelled glycosylated octreotate (Gluc-Lys-[^18^F]FP-TOCA) derivative. In a clinical study, this compound was compared with OctreoScan^®^ and demonstrated fast and high tumour uptake with rapid, predominately renal, excretion allowing observation of more than twice as many lesions as with OctreoScan^®^ [135]. The major drawback here was the complex and time-consuming production of the radiopharmaceutical, making a routine use in the clinic very difficult. [^18^F]-fluoroethyl triazole [Tyr^3^]-octreotate ([^18^F]FET-βAG-TOCA) [136] is another ^18^F-labelled octreotide derivative studied in humans. Synthesis is based on click chemistry and is an improvement compared to Gluc-Lys-[^18^F]FP-TOCA but the production remains time-consuming and complex (HPLC separation is necessary) with low radiochemical yield compared with ^68^Ga-labelling strategies. However, a first in human study showed rapid blood clearance with good tumour-to-background ratios, similar to [^68^Ga]-octreotide derivatives leading to further clinical trials, which are still ongoing [137]. A set of alternative labelling strategies including the introduction of aluminium mono-[^18^F]fluoride cations ([^18^F]AlF^2+^), labelling of SiFA by ^19^F–^18^F isotopic exchange reactions (IEX), and 1-step ^19^F–^18^F IEX on trifluoroborates such as trialkylammoniomethyl-BF_3_ are currently being evaluated (for a review, see [138]). Initial clinical trials with ^18^F-AlF-NOTA-octreotide [48] and ^18^F-SiFA*lin*-TATE [139] most recently published indicate promising data. Further studies will demonstrate if these compounds might replace the established ^68^Ga-labelled octreotide derivatives.

**Figure 1:**
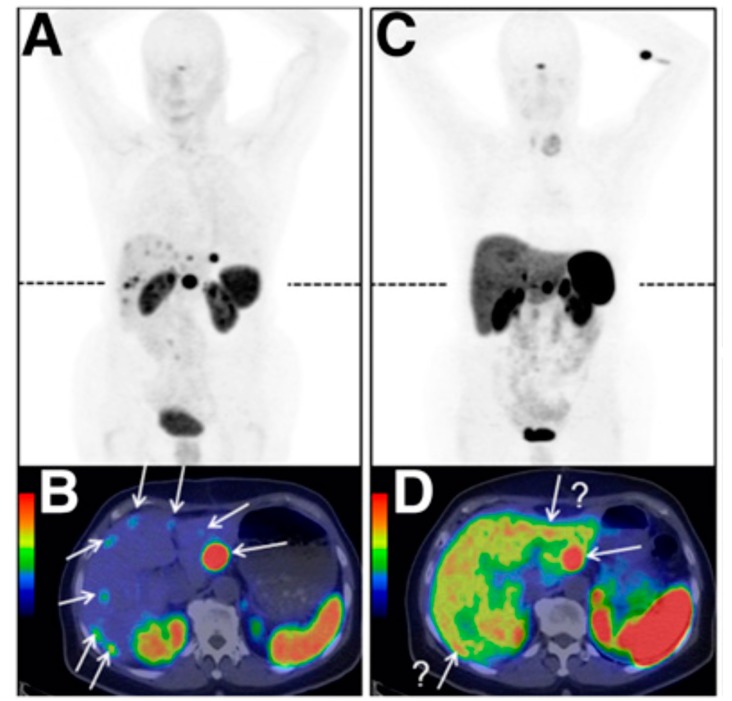
[^68^Ga]Ga-OPS202 PET/CT [antagonist—(**A**,**B**)] and [^68^Ga]Ga-DOTATOC PET/CT [agonist—(**C**,**D**)] images of a patient with ileal neuroendocrine tumours , showing bilobar liver metastases (dashed lines indicate level of transaxial slices). Studies were performed on the same scanner within 2 months and show same gray (maximal-intensity projections, (**A**,**C**) and colour scale (transaxial fusion images, (**B**,**D**). Importantly, background activity was lower in liver, intestine, and thyroid with [^68^Ga]Ga-OPS202 than with [^68^Ga]Ga-DOTATOC allowing better identification of the lesions in the liver which are confirmed by subsequent MRI. (Adapted from Nicolas et al., originally puplished in J Nucl Med [142]).

For a long time, it was a paradigm that internalisation of the radiopharmaceutical is a prerequisite for high tumour accumulation and especially necessary for the endoradiotherapy of tumours. Thus, the focus was on the development of SSTR agonists, which reveal high internalisation. Recently, H-Phe(pNO_2_)-cyclo(dCys-Tyr-dTrp-Lys-Thr-Cys)-dTyr-NH_2_ (BASS) [140] and H-Cpa-cyclo[dCys-Aph(Hor)-dAph(Cbm)-Lys-Thr-Cys]-dTyr-NH_2_) (JR11) [141] have been introduced. Both compounds are antagonists binding exclusively to SSTR-2, which does not internalise. Surprisingly, it could be demonstrated in murine tumour models that the antagonists have a higher tumour accumulation and retention than found for the corresponding agonist. These findings result in the hypothesis that an agonist that triggers a strong internalisation but binds to a limited number of high-affinity receptors might be a less-efficient targeting agent than an antagonist that lacks internalisation capabilities but binds to a larger variety of receptor conformations [140]. In an initial clinical study comparing the antagonist [^68^Ga]Ga-NODAGA-JR11 (also known as [^68^Ga]Ga-OPS202) and the agonist [^68^Ga]Ga-DOTATOC, it was found that the uptake (mean SUV_max_) in the lesions were comparable but the background, especially in the liver, was much lower for [^68^Ga]Ga-NODAGA-JR11, leading to much higher sensitivity [142] (see also Figure 1).

Octreotide derivatives labelled with yttrium-90 or lutetium-177 are used for the treatment of NETs (PRRT), including [^90^Y]Y-DOTATOC and [^177^Lu]Lu-DOTATATE [143]. In theory, depending on the physical properties of the different isotopes, [^90^Y]Y-DOTATOC should be used for the treatment of larger lesions and [^177^Lu]Lu-DOTATATE for smaller tumours. Nevertheless, in clinical praxis, [^177^Lu]Lu-DOTATATE is more frequently used than the ^90^Y-labelled analogue. Most recently, it was suggested that therapy protocols, including a combination of both radiopharmaceuticals, might be of advantage for the treatment of neoadjuvant and metastatic large volume NETs [143].

Studies show that a limitation of the [^177^Lu]Lu-DOTATATE therapy is that a large number of patients only achieve stabilisation of disease (26–55%) or are even refractory to β-radiation (18–32%) [144]. Thus, an alternative therapy strategy involves α-emitting isotopes for the treatment of NET. An initial clinical trial investigated the therapeutic effect of [^225^Ac]Ac-DOTATATE as an end-line treatment option [144]. A morphological response was assessed in 24/32 patients (15 partial remission; 9 stable disease). There was no documented disease progression or deaths in the median follow-up of 8 months and a significant decrease in the plasma chromogranin level post-therapy.

Meanwhile, SSTR antagonists are not only studied for diagnostic approaches but also for use as therapeutic radiopharmaceuticals. Dalm et al. [145] compared [^177^Lu]Lu-satoreotide tetraxetan ([^177^Lu]Lu-DOTA-JR11) and [^177^Lu]Lu-DOTATATE tracer accumulation in an in vitro cell uptake assay and the therapeutic effect in a murine tumour model and found 5-times higher cell uptake as well as 4.4-times higher tumour radiation dose for the antagonist. As expected for the antagonist, the majority of activity was membrane-bound and for the agonist, internalised. They concluded that SSTR antagonists might enhance PRRT and allow therapy for cancer types with relatively low receptor expression. An initial clinical trial with [^177^Lu]Lu-DOTA-JR11 indicates that this tracer delivers high radiation doses to the target structure with favourable tumour-to-normal organ dose ratios [146]. The therapeutic outcome is promising and supports that radiolabelled SSTR antagonists might be an alternative treatment strategy for NETs. However, for the antagonist, more severe hepatotoxicity was found during the treatment compared to agonist-based therapies, which might be reduced by modifications of the treatment protocols.

### 5.2. Prostate-Specific Membrane Antigen

Different studies revealed that the sequence Glu-C(O)-AA, with AA as a variable amino acid, is a potent inhibitor of glutamate carboxypeptidase II (GCPII) (see, e.g., [147]), an enzyme highly expressed on prostate carcinoma cells and also known as PSMA. Based on this finding, a variety of radiolabelled derivatives has been introduced, which have been labelled with different isotopes including iodine-125, technetium-99m, lutetium-177, actinium-225, fluorine-18 and gallium-68 (see, e.g., [85,148,149,150,151,152]). Although when accurately considered, these radiopharmaceuticals are more peptidomimetics than peptides, they were included here because many characteristics including labelling strategies are similar to those found for peptides. Meanwhile, a great diversity of compounds have been described (for a review, see [153]). Here we will focus on compounds studied in patients or already in clinical routine for diagnosis and therapy.

**Figure 2:**
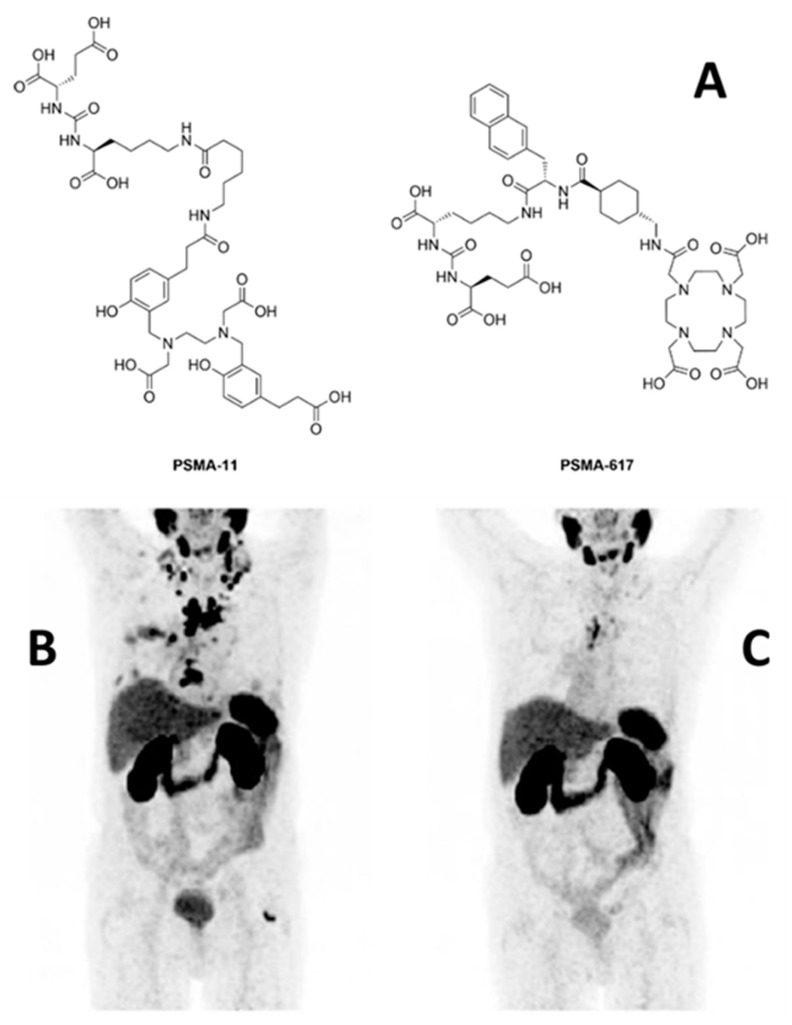
(**A**) Structure of Glu-C(O)-Lys(Ahx-HBED-CC) (PSMA-11) for labelling with gallium-68 and PSMA-617 for labelling with, e.g., gallium-68 and lutetium-177. (**B**) ^68^Ga-PSMA-11 PET at baseline and (**C**) after 2 cycles of [^177^Lu]Lu-PSMA-617 therapy demonstrating considerable reduction of PSMA-expressing metastases in lymph nodes and bone (adapted from Fendler et al., originally published in J Nucl Med [183]).

The big success of this class of radiopharmaceuticals is based on Glu-C(O)-Lys(Ahx-[^68^Ga]Ga-HBED-CC) (^68^Ga-PSMA-11) [85] (Figure 2), which was introduced for prostate carcinoma (PCa) diagnosis in 2011 [154]. The initial clinical study indicated that ^68^Ga-PSMA-11-directed PET imaging is significantly superior to alternative methods used for the detection of recurrent PCa [155], which is meanwhile confirmed by a large number of additional reports (for a review, see, e.g., [156,157]). Another ^68^Ga-labelled derivative used in clinical diagnostic of prostate cancer is [^68^Ga]Ga-PSMA-I&T [158]. In contrast to PSMA-11, the chelator 1,4,7,10-tetraazacyclododececane,1-(glutaric acid)-4,7,10-triacetic acid (DOTAGA) is used for complexation. Due to interaction with an additional arene-binding site within the PSMA structure, aromatic groups are necessary for high-affinity binding [159]. HBED-CC includes already aromatic residues whereas DOTAGA does not, which is why amino acids containing aromatic side chains are included in the spacer moiety to improve binding properties. The comparison of both tracers in patients revealed a higher sensitivity and therefore the slight superiority of ^68^Ga-PSMA-11 [160]. Other ^68^Ga-labelled compounds studied include [^68^Ga]Ga-THP-PSMA [161] and [^68^Ga]Ga-P16-093 [162]. Of advantage for [^68^Ga]Ga-THP-PSMA might be the rapid production via a kit labelling procedure and the low tracer accumulation in the body. Regardless of that, in an initial study uptake in tumour lesions was lower (as found for [^68^Ga]Ga-PSMA-I&T) but for a final concrete result, further studies where comparison of uptakes of both tracers in the same patient have to be carried out. [^68^Ga]Ga-P16-093, which differs from ^68^Ga-PSMA-11 only by a different linker moiety, demonstrated, in a pilot assessment, equivalent imaging properties in the detection of sites of PCa recurrence. Again, studies that are more comprehensive are needed.

Due to the increasing demand of PSMA/PET investigations, which is hardly satisfied by using ^68^Ge/^68^Ga-generators producing short-lived gallium-68, ^18^F-labelled compounds with a longer half-life enabling local distribution and lower positron energy, which might increase the resolution, have become a focus of interest. ^18^F-labelled PSMA targeting derivatives that are studied in patients include [^18^F]F-DCFBC [163], [^18^F]F-DCFPyL [164], [^18^F]F-PSMA-11 [165], [^18^F]F-PSMA-1007 [152], [^18^F]F-CTT1057 [166], and [^18^F]F-JK-PSMA-7 [167]. The first in-man studies have been carried out with [^18^F]F-DCFBC but these studies demonstrated slow clearance with high background activity [153]. An optimisation step where 4-[^18^F]fluorobenzyl-L-cysteine was replaced by 6-[^18^F]fluoronicotinoyl-L-lysine resulted in the more hydrophilic [^18^F]F-DCFPyL with much faster renal elimination [164]. In a direct comparison of [^18^F]F-DCFPyL and ^68^Ga-PSMA-11, the ^18^F-labelled radiopharmaceutical was found to perform equally well as the ^68^Ga-labelled reference [168]. Based on the binding motif of ^68^Ga-PSMA-11, [^18^F]F-PSMA-1007 was developed. In contrast to other derivatives, [^18^F]F-PSMA-1007 revealed low activity concentration in ureter and bladder but also higher background in the liver [169]. However, high imaging quality was found with both [^18^F]F-PSMA-1007 and [^18^F]F-DCFPyL, resulting in identical clinical findings in the evaluated routine situations. The low activity in the ureter is of advantage for [^18^F]F-PSMA-1007 in the delineation of local recurrence and the lower liver uptake of [^18^F]F-DCFPyL in cases where liver metastases occur [170]. Contrary to other PSMA targeting compounds, which are urea-based, [^18^F]F-CTT1057 is a phosphoramidate-based PSMA inhibitor. This compound resulted from a series of derivatives which differ in the linker between binding sequence and 4-[^18^F]fluorobenzoyl group [171]. A preliminary study with patients showed a low radiation burden as well as similar distribution as found for urea-based analogues like [^18^F]F-DCFPyL [166]. Efforts are made to optimise pharmacokinetics and detection rates of [^18^F]F-DCFPyL in patients with very low PSA levels. Therefore, different prosthetic groups were conjugated [167]. Out of this set, [^18^F]F-JK-PSMA-7 performed best and was further evaluated in patients. In this clinical setting, [^18^F]F-JK-PSMA-7 showed no drug-related adverse effects but also revealed no inferior sensitivity in detecting prostate cancer lesions, compared to ^68^Ga-PSMA-11 [172]. In a few selected patients, [^18^F]F-JK-PSMA-7 revealed superior sensitivity compared with the lead structure [^18^F]F-DCFPyL in detecting PSMA-positive lesions in small lymph nodes. Another approach is based on the use of [^18^F]AlF for labelling of PSMA-11. An automated labelling process has been validated [173] and an initial clinical study to evaluate the safety of administration and radiation dosimetry has been performed [174]. Here, [^18^F]F-PSMA-11 could be safely administered with a mean effective dose comparable with [^18^F]F-DCFPyL. The ability to detect PSMA-positive lesions and the performance compared to other PSMA-targeting tracer needs to be further explored.

Based on the successful introduction of the diagnostic radiopharmaceuticals, derivatives allowing endoradiotherapy have also been developed. Because HBED-CC is not optimal for the complexation with therapeutically used α- and β-emitter like actinium-225 and lutetium-177, this moiety had to be replaced. As chelator of choice DOTA and its derivatives have been introduced. As indicated for high binding affinity and receptor internalisation, aromatic moieties interacting with the arene-binding pocket would be of advantage; thus, spacers containing aromatic groups are included. The most used β-emitting radiopharmaceuticals are [^177^Lu]Lu-PSMA-617 [151] and [^177^Lu]Lu-PSMA-I&T [158]. A retrospective multicentric clinical trial including more than 140 patients demonstrated the favourable safety and efficacy of [^177^Lu]Lu-PSMA-617 therapies [175]. Additional prospective clinical trials are ongoing [e.g., TheraP (NCT03392428) and VISION (NCT03511664)] to evaluate the potential of this endoradiotherapy, especially with regard to improved patient survival. Another study including 100 patients treated with [^177^Lu]Lu-PSMA-I&T showed that this tracer is also well-tolerated and demonstrated good treatment response in a subgroup of patients [176]. It was also observed that treatment outcome was worse in patients with organ metastases and elevated lactate dehydrogenase in blood tests. In the most recent approaches, albumin binders are suggested for endoradiotherapy to modify pharmacokinetics and to enhance therapeutic efficacy [177]. Evans blue (EB) is one compound used for this purpose; thus, [^177^Lu]Lu-EB-PSMA-617 has been introduced [178], which revealed in a first in human study [179] higher accumulation in metastatic castration-resistant prostate cancer (mCRPC) than [^177^Lu]Lu-PSMA-617 and that a lower dose of the new radiopharmaceutical appears to be already effective in treating tumours. The elevated uptake of [^177^Lu]Lu-EB-PSMA-617 in kidneys and red bone marrow seems to be well tolerated despite that studies which are more comprehensive are necessary to assess the potential of this approach.

As discussed, ^177^Lu-labelled PSMA enzyme inhibitors like [^177^Lu]Lu-PSMA-617 and [^177^Lu]Lu-PSMA I&T have favourable dosimetry and convincing therapeutic response but approximately one-third of patients were found to respond only for a short period or were non-responders. Moreover, problematic is that dose escalation is limited by chronic haematological toxicity [180]. Based on these limitations, α-emitting radiopharmaceuticals have been introduced. The isotopes included are astatine-211, bismuth-213, and actinium-225, but so far only PSMA-617 labelled with bismuth-213 or actinium-225 were studied in clinical trials. In a case report, a patient who was progressive under conventional therapy showed remarkable response 11 months after two cycles of [^213^Bi]Bi-PSMA-617 [181]. The α-emitting [^225^Ac]Ac-PSMA-617 appeared to have high clinical efficacy when compared to β-emitting therapy with [^177^Lu]Lu-PSMA-617 [182]. However, α-emitting therapy resulted in a higher radiation dose for the salivary glands, which is why the authors suggest modifications of the treatment regimen to overcome this problem.

### 5.3. Integrins

**Figure 3:**
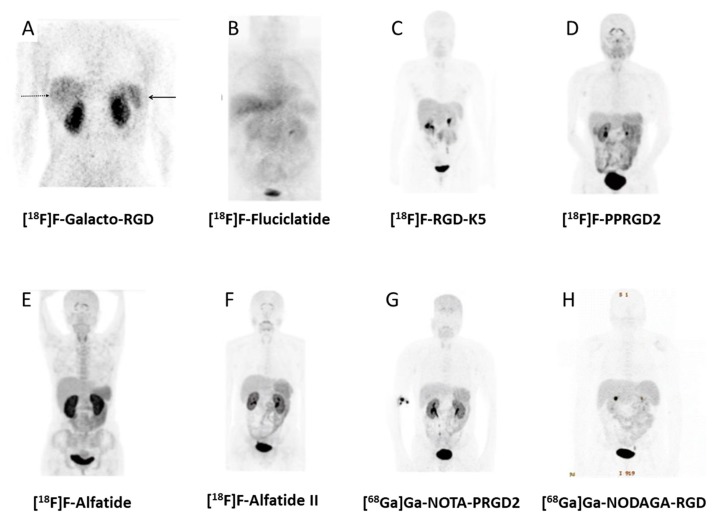
PET images of some clinically studied RGD-based tracers 1 h after intravenous administration in healthy volunteers (**B**–**D**,**F**), a patient with melanoma (**A**), scrofula (**E**), lung cancer (**G**), and hepatocellular carcinoma (**H**). All images are coronal views. High tracer retention is notable in urogenital tract, due to predominant renal clearance. Intermediate uptake is found in the liver, spleen, and intestines. Reproduced with the permission from [5,45,47,190,207,208,209,210].

Already some decades ago, great hope has been pinned on the “starvation” of tumours by inhibition of tumour-induced angiogenesis. One target structure involved in these processes is the integrin α_v_β_3_; thus, a great variety of radiopharmaceuticals have been developed to target this receptor to allow treatment planning and control of corresponding antiangiogenic therapies (for a review, see, e.g., [4,184,185]). Most of these peptides are based on the cyclic pentapeptide structure c(RGDfV) developed by Kessler’s group in 1991 [186]. Already, the initial compounds labelled with iodine-125 demonstrated receptor specific tumour accumulation [187]. In subsequent investigations, optimisations improving pharmacokinetics, tracer uptake, and target retention were performed, resulting in a comprehensive set of radiopharmaceuticals of which several have been studied in patients. The first and most intensive evaluated compound in humans has been [^18^F]F-Galacto-RGD [11,188]. The initial clinical trial demonstrated receptor-specific accumulation in integrin α_v_β_3_ positive tissue and rapid renal elimination with low background activity in most tissue [189]. Further studies confirmed these findings and revealed that the radiopharmaceutical is well tolerated with an effective dose comparable with an [^18^F]FDG-PET scan [190,191,192]. Based on these results, alternative ^18^F-labelled compounds have been examined in clinical trials including [^18^F]F-RGD-5K, [^18^F]F-Fluciclatide, [^18^F]FPPRGD2, [^18^F]F-Alfatide, and [^18^F]F-Alfatide II. [^18^F]F-RGD-5K is also based on c(RGDfK) conjugated to a sugar moiety but labelling is done via click chemistry, reducing the production time to 70 min [193]. For the synthesis of [^18^F]F-Fluciclatide, a disulfide bridged peptide structure, resulting from a phage display library and demonstrating higher binding affinity for α_v_β_5_ as for α_v_β_3_, is used [194]. Labelling was carried out via oxime formation but did not reduce production time dramatically compared to [^18^F]F-Galacto-RGD. Initially, Wester’s group demonstrated that combining more than one RGD binding epitope in one molecule improved binding affinity [31,195]. This multimerisation approach was applied for [^18^F]F-PPRGD2 [196] as well as for [^18^F]F-Alfatide [197] and [^18^F]F-Alfatide II [46], which contain two binding epitopes. Labelling of FPPRGD2 was carried out via [^18^F]-fluoropropionic acid as a prosthetic group. Alfatide and Alfatide II, which differ in the design of the spacer/branching unit, making the latter more stable, were labelled using [^18^F]aluminium fluoride. With exception of the [^18^F]aluminium fluoride labelled derivatives, all other ^18^F-labelled compounds are characterised by complex synthesis routes including at least one HPLC separation step. To overcome this drawback, ^68^Ga-labelled alternatives have been suggested. Three of these derivatives, namely [^68^Ga]Ga-NOTA-RGD [118], [^68^Ga]Ga-NODAGA-RGD [86], and [^68^Ga]Ga-NOTA-PRGD2, [197] were tested in clinical trials (examples of PET images of the different RGD-based tracers in clinical studies are presented in Figure 3).

Although the structure and label of the clinically studied RGD-tracers differ, the in vivo pharmacokinetic properties are comparable with predominant renal elimination and prominent uptake in kidneys and bladder. Thus, initial voiding is recommended before starting the PET/CT scan. Additional increased activity concentration was found in the liver, spleen, and intestines, which is why optimal regions for lesion detection are suggested to be lungs, mediastinum, head-and-neck area, thorax including the breast, skeletal system and the extremities [5,184]. Because the RGD-peptides do not cross the intact blood–brain-barrier, background in brain is very low, allowing easy identification of brain tumours even if tumour uptake is low (see, e.g., [198]). Identification of lesions in the body differs inter- as well as intra-individual [190]. A great diversity of different tumour entities including melanoma, sarcoma, glioblastoma, non-small cell lung cancer, breast cancer, rectal cancer, bone metastasis, and renal cell cancer (for a review, see [184]) have been studied. Due to differences in the imaging protocols and low patient numbers, final conclusions are difficult to draw. However, sensitivity in lesion detection was over 90% for sarcoma, non-small cell lung cancer, glioblastoma, renal cell cancer, breast cancer, and some bone metastases. Moreover, where immunohistochemical staining of corresponding tumour tissue was possible, a correlation with receptor expression was found [191].

A variety of preclinical studies have demonstrated the potential to monitor response to antiangiogenesis therapies in murine tumour models [199,200,201,202,203]. All studies, using different antiangiogenic pharmaceuticals including Endostar, sunitinib, ZD4190, linifanib, and dasatinib, already showed a reduction of uptake within 3 days after treatment start, where tumour size was only slightly affected and [^18^F]FDG did not reveal significant changes in uptake. Translation into clinics is rather difficult and only a few clinical studies have been published. A pilot study comparing therapy control of a bevacizumab-containing therapy using [^18^F]-FPPRGD2 or [^18^F]FDG, respectively, showed a decrease of tumour uptake of [^18^F]-FPPRGD2 early after therapy start [204]. Another clinical trial included 38 patients with solid malignancies and studied the predictive value of [^18^F]F-Alfatide before an antiangiogenic therapy with apatinib was started [205]. It was demonstrated that lesions with higher tracer uptake showed better response to the apatinib therapy. These initial data are promising but more comprehensive studies that clearly demonstrate that the therapeutic effects of anti-angiogenic therapies can be predicted and controlled are necessary for the successful translation of this tracer into clinical routine.

It has to be mentioned that not only radiopharmaceuticals targeting the integrin α_v_β_3_/α_v_β_5_ are developed but also compounds targeting other integrins including α_v_β_6_ and α_5_β_1_ (for a review, see, e.g., [4,206]. Until now, to our knowledge, none of these compounds have entered clinical trials and will not be discussed here.

### 5.4. Chemokine Receptor

**Figure 4:**
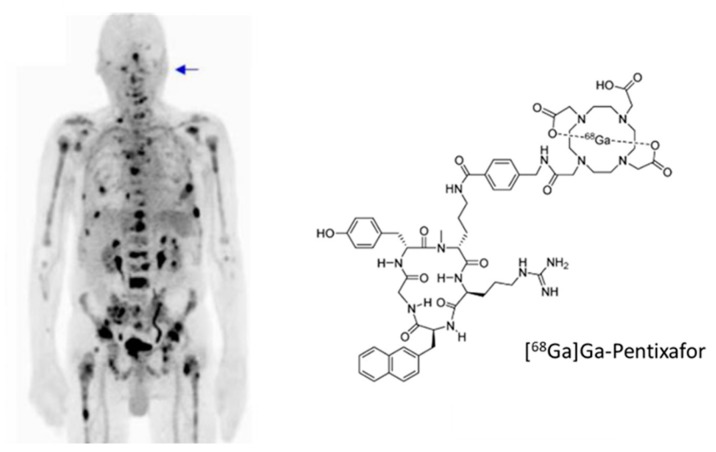
Structure of [^68^Ga]Ga-Pentixafor and PET MIP of a patient with multiple myeloma (modified from Wester et al., originally published in Theranostics [226]).

The chemokine receptor-4 CXCR4 and its ligand CXCL12 play an important role in tumour development and metastasis and are found in breast, prostate, lung, colorectal and brain tumours (for a review, see, e.g., [211]). This receptor mediates organ-specific metastasis and the level of expression was found to be predictive for the metastatic potential of the tumours [212]. Due to the central role in tumour development, a variety of inhibitors of the CXCR4/CXCL12 interaction has been developed during the last decade [213,214]. Based on these compounds, radiolabelled analogues for non-invasive determination of the CXCR4 expression have been evaluated [211]. They are based either on the bicyclams AMD3100 and AMD3465, on the disulfide-bridged peptide T-140, or on the cyclic pentapeptide FC-131. So far, only [^68^Ga]Ga-NOTA-NFB and [^68^Ga]Ga-Pentixafor have been included in clinical studies. [^68^Ga]Ga-NOTA-NFB was studied in healthy volunteers and a limited number of glioma patients [215]. The compound was well tolerated with highest activity accumulation found in the liver, spleen, kidneys, and bladder. The low background in the brain and the receptor specific accumulation in the tumour suggest further investigations to confirm the potential of diagnosing and evaluating glioma patients. More extensive clinical studies have been carried out with [^68^Ga]Ga-Pentixafor (Figure 4) (for an overview, see, e.g., [211]). However, accumulation in solid cancer and metastases seems not as high as expected from in vitro CXCR4 expression profiles [216]. Thus, the detectability of solid cancer using [^68^Ga]Ga-Pentixafor seems to be inferior, as found for [^18^F]FDG. This is confirmed by a most recent study, which concludes that “CXCR4-directed imaging may not play a major role in the management of solid tumours in the majority of patients” [217]. In contrast, studies including hematologic malignancies where high CXCR4 expression is found, like non-Hodgkin-lymphoma, multiple myeloma, chronic lymphocytic leukaemia and acute myeloid leukaemia, depict the potential of [^68^Ga]Ga-Pentixafor PET/CT as a diagnostic marker for these malignancies [211]. [^68^Ga]Ga-Pentixafor for PET/CT was able to image disease manifestation in 10/14 patients with myeloid leukaemia [218] and in 23/34 patients in another study [219]. Further evaluations showed the applicability of [^68^Ga]Ga-Pentixafor PET/CT to identify patients with CXCR4-positive acute myeloid leukaemia [220] and chronic lymphocytic leukaemia [221]. It is assumed that CXCR4-directed imaging might be more included in patient selection for corresponding therapeutic approaches rather than to evaluate disease extent.

In another approach, Pentixafor was modified allowing the use for endoradiotherapy with lutetium-177 and yttrium-90. The resulting [^177^Lu]Lu/[^90^Y]Y-Pentixather [222] was studied in patients with advanced stage multiple myeloma [223], diffuse large B cell lymphoma [224], and acute myeloid leukaemia [225]. Initial data are promising, but extended studies are needed to evaluate the potential of this new treatment option.

### 5.5. Other Target Structures Expressed on Tumour Cells

Beside the above-mentioned radiopharmaceuticals and their corresponding target structures, a variety of other radiolabelled compounds are used for the non-invasive determination of a set of receptors overexpressed on tumour cells. These include CCK2-R, the gastrin-releasing peptide receptors (GRP-R), the uPAR, the glucagon-like peptide receptor 1 (GLP-1), melanocortin 1 receptor, neurotensin receptor, neurokinin 1 receptor (NK_1_-R), neuropeptide Y receptor (NPY-R), the vasoactive intestinal peptide receptors (VPAC1 and 2), and caspase-3 (for an overview, see, e.g., [123,124]). To our knowledge, only radiopharmaceuticals targeting CCK2-R, GRP-R, uPAR, GLP-1, NK_1_-R, VPAC1, and caspase-3 are investigated in patients so far. Due to the great diversity of different compounds and to not go beyond the scope of this review, we will give only a brief summary here.

A great variety of NK_1_ targeting radiopharmaceuticals has been developed and studied in initial clinical trials (for a review, see [227]). However, PET tracer development strongly focused on the diagnosis of central nervous system related diseases and mainly uses small molecule antagonists of the receptor as lead structures. Diagnostic tracers in oncology are mainly described for SPECT applications and are derivatives of substance P. Moreover, there are different compounds labelled with bismuth-213, actinium-225, lutetium-177 and yttrium-90 for tumour treatment with the major focus on local administration to cure glioblastoma (see, e.g., [228]).

The value of radioiodinated VIP for peptide receptor scintigraphy of carcinoid patients was already explored in the 1990s [229]. Because VPAC1 is highly expressed on prostate cancer cells more recently, 25 patients undergoing radical prostatectomy were imaged with PET/CT preoperatively with [^64^Cu]Cu-TP3805, and the tracer allowed delineation of prostate carcinoma and is worthy of further studies [230]. Similar findings are described for patients with breast cancer [231].

Most attempts to image CCK2-R expression are based on minigastrin derivatives labelled with indium-111 or technetium-99 m for SPECT (for a review, see [232]). Up to now, only one patient was investigated with [^68^Ga]Ga-DOTA-MG0 for PET imaging, revealing increased uptake in the right thyroid with physiological uptake in the stomach, together with highly reduced uptake in the liver, spleen and kidneys compared to [^68^Ga]Ga-DOTA-TATE[233]. Already in 2002, [^90^Y]Y-DTPA-MG0 was studied in 8 patients [234] with limited therapeutic success and severe nephrotoxic side effects, a reason why this study was not continued. Recently, pharmacokinetic properties, dosimetry, and the targeting ability of [^177^Lu]Lu-DOTA-PP-F11N was tested in an initial clinical study for potential use of CCK2 receptor-based endoradiotherapy [235]. Tumour uptake seems sufficient for successful therapeutic use, with the stomach as the main dose-limiting organ.

Caspase-3 is a cysteine protease playing a central role during apoptosis [236] and is of interest as a target structure to monitor therapies aimed to activate the apoptotic pathway [124]. The ^18^F-labelled pentapeptide CP-18 is a substrate of caspase-3, which showed increased activity uptake in apoptotic thymi in mice [237]. Based on these promising findings, [^18^F]F-CP-18 was studied in seven healthy volunteers [238]. The images showed uptake in the liver, heart, testes, kidneys, and bladder and due to the predominantly renal excretion, the critical organ in terms of dosimetry was the bladder wall.

The GRP-R family consists of four receptors (BBR1-4) of whom three are expressed in humans. Especially, BBR1 and BBR2 are upregulated in cancer cells, including breast, colon, lung, pancreatic, and prostate cancers [239]. An initial lead structure for tracer development was found in the 14 amino acids containing bombesin (BBN) [240]. Corresponding compounds revealed low stability in humans; thus, further development was focused on the C-terminal sequence containing amino acid 7 to 14 of BBN and introduced a variety of sequence modifications to improve the metabolic stability (see review [124]). One of the first PET tracers in clinical studies was [^68^Ga]Ga-DOTA-PEG_2_-[DTyr^6^-β-Ala^11^,Thi^13^,Nle^14^]BBN(6-14) amide ([^68^Ga]Ga-BZH3). It was mainly investigated in patients with glioma, where it showed superior tumour grading in comparison to [^18^F]FDG [241]. Another peptide that entered clinical trials is DO3A-CH_2_CO-G4-aminobenzyl-Gln-Trp-Ala-Val-Gly-His-Leu-Met-NH_2_ (AMBA), which was initially labelled with lutetium-177 and subsequently with gallium-68 [124]. However, detailed data concerning these studies are not yet available. As already discussed for SSTR targeting, radiopharmaceuticals antagonists are becoming more popular and have also been developed to target GRP-R with superior properties and reduced side effects. An initial clinical study using [^64^Cu]4,11-bis(carboxymethyl)-1,4,8,11-tetraazobicyclo(6.6.2)hexadecane-PEG_4_-dPhe-Glu-Trp-Ala-Val-Gly-His-Sta-Leu-NH_2_ ([^64^Cu]Cu-CB-TE2A-AR06) in prostate cancer patients showed favourable biodistribution with low activity concentration in kidneys and intestine but high tumour uptake [242]. Another BBN antagonist was developed by Maina et al. [243] and studied in prostate and breast cancer patients. Uptake of [^68^Ga]Ga-SB3 was found in approximately 50% of the studied patients. Further tracers studied in patients are NeoBomb1 and [^68^Ga]Ga-BAY86-7548/[^68^Ga]Ga-RM2. First in-man studies with [^68^Ga]Ga-NeoBomb1 in prostate cancer patients showed that the compound is well tolerated and that primary, as well as multimetastic foci, could be detected [6]. Additional evaluations are ongoing to demonstrate the potential of the tracer. [^68^Ga]Ga-BAY86-7548/[^68^Ga]Ga-RM2 was also studied in prostate cancer patients [244] but also included breast cancer patients [245] in a subsequent study. Moreover, the tracer was compared in a head-to-head study with ^68^Ga-PSMA-11 in patients with biochemically recurring prostate cancer [246]. In this study, ^68^Ga-PSMA-11 performed superior to [^68^Ga]Ga-BAY86-7548/[^68^Ga]Ga-RM2, but findings showed similar uptake between the two tracers in suspected lesions.

The uPAR is a membrane-anchored receptor for the corresponding plasminogen activator. This serine protease is activated if bound to the receptor and cleaves plasminogen to plasmin that activates proteolytic processes on the extracellular matrix [247]. Due to the overexpression on breast, prostate, pancreatic, and colorectal cancer, where it was found to correlate with tumour aggressiveness, it has become an attractive target structure for the development of PET tracers [124]. One compound studied in patients with breast, prostate, and bladder cancers is [^64^Cu]Cu-DOTA-AE105 [248] which was well tolerated and showed rapid renal elimination. Despite low plasma half-life, primary tumours in breast and prostate cancer patients could be detected. Subsequently, the chelator DOTA was replaced by NOTA and [^68^Ga]Cu-NOTA-AE105 was studied in patients with the same tumour entities where comparable results as for the initial compound were found [249].

The GLP-1 receptor is expressed on beta cells and is an ideal target structure for the non-invasive determination of the beta-cell mass [124]. The receptor is involved in the regulation of the balance between glucose and insulin levels but is also expressed on insulinoma [250]. The endogenous glucagon-like peptide 1 has a very short half-life and can hardly be used for imaging [251]. Exendin-4, isolated from the saliva of the Gila monster *Heloderma suspectum,* also binds to this receptor and demonstrated higher stability [252]. Thus, [Nle^14^-Lys^40^-(Ahx-DOTA-^68^Ga)-NH_2_]exendin-4 ([^68^Ga]Ga-DOTA-exendin-4) was developed and compared with the ^111^In-labelled analogue in five patients with insulinoma. The study revealed that the tracer allows the delineation of tumour foci with a higher tumour/background ratio for the ^68^Ga-labelled derivative [253]. The superiority of [^68^Ga]Ga-DOTA-exendin-4 is confirmed by a more comprehensive trial including 52 patients [254]. [^68^Ga]Ga-NOTA-MAL-Cys40-exendin-4 ([^68^Ga]Ga-NOTA-exendin-4) is another compound studied in patients with insulinomas [255]. The [^68^Ga]Ga-NOTA-exendin-4 PET/CT showed a sensitivity of 97.7%, which is significantly higher than for commonly used imaging strategies. Most recently, dosimetry calculations for [^68^Ga]Ga-NODAGA-exendin-4 have been presented, indicating low radiation burden of the corresponding PET/CT investigations [256].

Recently, fibroblast activation protein inhibitors (FAPI) have become a focus of interest because this transmembrane serine protease is involved in the development of a great variety of tumour types [257] and initial clinical studies have demonstrated very promising results with high tumour uptake and good tumour/background ratios [258]. Most of the studied ^68^Ga-labelled radiopharmaceuticals are based on a Gly-Pro unit, which is found to be a binding motive of this protease. However, these quinolone-based compounds [259] are seen more as small organic molecules and are not in the scope of this review.

## 6. Summary and Conclusions

Due to the central role of peptide/receptor interactions, the development of radiolabelled probes for the use in nuclear medicine tracer techniques has been of interest for several decades. Besides the broad applicability, the high diversity of labelling options and approaches to optimise the pharmacokinetic properties makes this class of tracer attractive for the development of radiopharmaceuticals for diagnosis and therapy of oncological diseases. As described above, even the problematic low metabolic stability of a variety of lead compounds can be overcome by various ways of modifications. This led to the development of radiopharmaceuticals for a diversity of target structures including SSTR, integrins, PSMA, chemokine receptor-4, CCK2-R, GRP-R, uPAR, and GLP-1. Radiopharmaceuticals binding to SSTR and PSMA are already in clinical routine for the diagnosis and treatment of neuroendocrine tumours and prostate cancer, respectively. Others are being studied in clinical trials and their outcomes have to demonstrate their potential.

For PET diagnosis, gallium-68 was the best choice of isotope, but due to the limited activity provided from corresponding generators, there has been a renaissance in the development of ^18^F-labelled analogues. Despite the improvement of the accessibility of corresponding prosthetic groups especially for remote controlled production processes, further developments might be needed. An alternative labelling strategy introduces [^18^F]-aluminium fluoride, which allows straightforward labelling involving chelator systems in analogy to radiometals. However, the potential of this strategy has not yet been demonstrated in routine clinical use. For labelling of proteins and antibodies, zirconium-89 has come into focus because its longer half-life fits better to the elimination kinetics of these molecules. For small peptides, this might be of no great benefit. In PRRT, the β-emitters yttrium-90 and lutetium-177 are the isotopes mainly used. Recently, α-emitting isotopes like astatine-211 and actinium-225 were also investigated and demonstrated in initial studies some positive results compared to therapies with β-emitters.

For a long time, it was thought that internalising agonists are superior to non-internalising antagonists, but recent studies with radiolabelled antagonists of SSTR-2 revealed contrary results and initiated a paradigm shift. Meanwhile, first antagonists for diagnosis and therapy of NET are investigated in clinical trials, and for other receptors like the GRP-R, antagonists are under development.

Altogether, peptide-based radiopharmaceuticals are important tools in molecular imaging using PET as well as theranostics. Besides the established tracer for the diagnosis and therapy of NET and PCa, several others are currently being evaluated and some of them might soon be used in clinical routine (Table 3). Moreover, due to the comprehensive research in developing new labelling strategies as well as in the optimisation of corresponding peptides, the development of peptide-based tracers will remain a central field in radiopharmaceutical research.

## Figures and Tables

**Table 1:** Selected physical parameters of the discussed isotopes for positron emission tomography (PET) imaging and therapy (available from The Lund/LBNL Nuclear Data Search: http://nucleardata.nuclear.lu.se/toi/; Laboratoire National Henri Becquerel: http://www.lnhb.fr/nuclear-data/nuclear-data-table/).

Isotope	Half-Life [h]	Decay Mode	Energy (keV)
Halogens			
[^18^F]	1.82	β^+^ (96.9%) EC ^a^ (3.1%)	β^+^: 633.9
[^76^Br]	16.2	β^+^ (54%) EC ^a^ (46%)	β^+^: 4963
[^124^I]	4.176 d	β^+^ (23%) EC ^a^ (77%)	β^+^+EC ^a^: 3160
[^131^I]	8.0233 d	β^−^ (100%)	β^−^: 970.8
[^211^At]	7.22	α (41.8%) EC ^a^ (58.2%)	α: 4000–8500
Radiometals			
[^64^Cu]	12.7	β^+^ (17.8%) β^-^ (38.4%) EC ^a^ (43.5%)	β^+^: 653 β^−^: 579
[^68^Ga]	1.1	β^+^ (88.9%) EC ^a^ (11.1%)	β^+^: 1899.1
[^89^Zr]	78.42	β^+^ (23%) EC ^a^ (77%)	β^+^: 902
[^90^Y]	64.1	β^−^ (100%)	β^−^: 2279
[^177^Lu]	159.4	β^−^ (100%)	β^−^: 498
[^225^Ac]	240	α (100%)	α: 5800–8400

^a^ Electron capture.

**Table 2:** Summary of chelators for the different radioactive isotopes and published labelling conditions (reaction temperature and incubation time).

Isotope	Chelator	Labelling Conditions	Reference
[^64^Cu]	CB-TE2A ^a^	95 °C, 1 h	[113]
	CB-TE2P	RT	[80]
	DOTA ^b^	RT, 15 min	[114]
	NOTA ^c^	RT, 45 min	[115]
	PCB-TE2A	RT	[75]
	TETA ^d^	RT, 45 min	[74]
	Sarcophagine-based chelators	RT, within min	[75,82]
[^68^Ga]	AAZTA ^e^	RT, 10 min	[116]
	DFO ^f^	RT, 5 min	[116]
	DOTA ^b^	95 °C, 5 min	[116]
	DTPA ^g^	RT, 10 min	[117]
	HBED-CC ^h^	80–100 °C, 5–20 min	[85]
	NODAGA ^i^	RT, 10 min	[86]
	NOTA ^c^	RT, 10 min	[118]
	TRAP^j^/NOPO ^k^	95–100 °C, 5 min; RT or 95 °C, 5 min	[87,89]
[^89^Zr]	DFO ^f^	RT, 30 min	[119]
	DOTA ^b^	95 °C, 60 min	[97]
	FSC ^l^	RT, 90 min	[120]
[^90^Y]	3p-C-NETA-NCS ^m^	RT, 60 min	[106]
	CHX-A’’-DTPA ^n^	37–75 °C, 30–60 min	[82]
	DOTA ^b^	80 °C, 20 min	[121]
[^177^Lu]	3p-C-NETA-NCS ^m^	RT, 60 min	[106]
	AAZTA-5	RT, 10 min	[104]
	DOTA ^b^	80 °C, 20 min	[121]
	H_4_octapa ^o^	RT, 15 min	[105]
[^224^Ac]	DOTA ^b^	95 °C, 5 min	[109]
	H_2_macropa ^p^	RT, within several min	[112]
	HEHA ^q^	37 °C, 30 min	[122]

^a^ 4,11-bis(carboxymethyl)-1,4,8,11-tetraazabicyclo[6.6.2]hexadecane, ^b^ 1,4,7,10-tetraazacyclododecane-1,4,7,10-tetraacetic acid, ^c^ 1,4,7-triazacyclononane-1,4,7-triacetic acid, ^d^ 1,4,8,11-tetraazacyclotetradecane-1,4,8,11-tetraacetic acid, ^e^ 1,4-bis(carboxymethyl)-6-[bis(carboxymethyl)]amino-6-methylperhydro-1,4-diazepine, ^f^ desferrioxamine B, ^g^ diethylenetriamine pentaacetic acid, ^h^ *N,N′*-bis[2-hydroxy-5-(carboxyethyl)benzyl]ethylenediamine-*N,N′*-diacetic acid, ^i^ 1,4,7-triazacyclononane,1-gluteric acid-4,7-acetic acid, ^j^ 1,4,7-triazacyclononane phosphinic acid, ^k^ 1,4,7-triazacyclononane-1,4-bis[methylene(hydroxymethyl)phosphinic acid]-7-[methylene(2-carboxyethyl) phosphinic acid], ^l^ fusarinine C, ^m^ {4-[2-(Bis-carboxy-methylamino)-5-(4-isothiocyanatophenyl) pentyl]-7-carboxymethyl[1,4,7] triazonan-1-yl}acetic acid, ^n^ 2-(p-isothiocyanatobenzyl)-cyclohexyldiethylenetriamine pentaacetic acid, ^o^ *N,N′*-bis(6-carboxy-2-pyridylmethyl)-ethylenediamine-*N*,*N*′-diacetic acid), ^p^ *N*,*N*’-bis[(6-carboxy-2-pyridil)methyl]-4,13-diaza-18-crown-6, ^q^ 1,4,7,10,13,16-hexaazacyclohexadecane-*N*,*N*′,*N*″,*N*‴,*N*′‴,*N*″‴-hexaacetic acid.

**Table 3:** Peptides used in clinical trials or clinical routine PET diagnosis.

Peptide/Chelator	Isotope	Target Receptor	Tumour Types	Reference
AlF-NOTA-octreotide	[^18^F]	SSTR ^a^	NET ^b^	[48]
DOTA-JR11 (OPS201)	[^177^Lu]	SSTR ^a^ (antagonist)	NET ^b^	[145]
DOTANOC	[^68^Ga]	SSTR-2/3/5 ^a^	NET ^b^	[127]
DOTATATE	[^68^Ga]	SSTR-2 ^a^	NET ^b^	[125]
	[177Lu]/[90Y]			[143]
	[64Cu]			[132]
	[225Ac]			[144]
DOTATOC	[^68^Ga]	SSTR-2/5 ^a^	NET ^b^	[1]
	[90Y]			[143]
FET-βAG-TOCA	[^18^F]	SSTR ^a^	NET ^b^	[136]
Gluc-Lys-[^18^F]FP-TOCA	[^18^F]	SSTR ^a^	NET ^b^	[134]
NODAGA-JR11 (OPS202)	[^68^Ga]	SSTR-2 ^a^ (antagonist)	NET ^b^	[142]
SiFA*lin*-TATE	[^18^F]	SSTR ^a^	NET ^b^	[139]
TETA-octreotide	[^64^Cu]	SSTR-2 ^a^	NET ^b^	[131]
CTT1057	[^18^F]	PSMA ^c^	prostate cancer	[166]
DCFPyL	[^18^F]	PSMA ^c^	prostate cancer	[164]
EB-PSMA-617	[^177^Lu]	PSMA ^c^	prostate cancer	[178]
HBED-CC-PSMA (PSMA 11)	[^68^Ga]	PSMA ^c^	prostate cancer	[85]
	[18F]			[165,174]
JK-PSMA-7	[^18^F]	PSMA ^c^	prostate cancer	[167]
PSMA-I&T	[^68^Ga]	PSMA ^c^	prostate cancer	[158]
	[177Lu]			[176]
PSMA 617	[177Lu]	PSMA ^c^	prostate cancer	[151]
	[213Bi]			[181]
	[225Ac]			[182]
P16-093	[^68^Ga]	PSMA ^c^	prostate cancer	[162]
PSMA-1007	[^18^F]	PSMA ^c^	prostate cancer	[152]
THP-PSMA	[^68^Ga]	PSMA ^c^	prostate cancer	[161]
Alfatide	[^18^F]	integrin α_v_β_3_	tumour angiogenesis	[197]
Alfatide II	[^18^F]	integrin α_v_β_3_	tumour angiogenesis	[46]
Fluciclatide	[^18^F]	integrin α_v_β_3_	tumour angiogenesis	[194]
FPPRGD2	[^18^F]	integrin α_v_β_3_	tumour angiogenesis	[196]
Galacto-RGD	[^18^F]	integrin α_v_β_3_	tumour angiogenesis	[11,188]
NODAGA-RGD	[^68^Ga]	integrin α_v_β_3_	tumour angiogenesis	[5]
NOTA-RGD	[^68^Ga]	integrin α_v_β_3_	tumour angiogenesis	[118]
NOTA-PRGD2	[^68^Ga]	integrin α_v_β_3_	tumour angiogenesis	[197]
RGD-5K	[^18^F]	integrin α_v_β_3_	tumour angiogenesis	[193]
NOTA-NFB	[^68^Ga]	chemokine receptor-4	glioblastoma	[215]
Pentixafor	[^68^Ga]	chemokine receptor-4	glioblastoma	[211]
Pentixather	[^177^Lu] [^90^Y]	chemokine receptor-4	multiple myeloma, diffuse large B cell lymphoma, acute myeloid leukemia	[222]
TP3805	[^64^Cu]	VPAC1 ^d^	prostate cancer	[230]
DOTA-MG0	[^68^Ga]	CCK2-R ^e^	MTC ^f^	[233]
DTPA-MG0	[^90^Y]	CCK2-R ^e^	MTC ^f^	[234]
DOTA-PP-F11N	[^177^Lu]	CCK2-R ^e^	MTC ^f^	[235]
CP-18	[^18^F]	caspases	apoptosis	[237,238]
CB-TE2A-AR06	[^64^Cu]	bombesin	prostate cancer	[242]
BAY86-7548/RM2	[^68^Ga]	bombesin	prostate/breast cancer	[244,245]
BZH_3_	[^68^Ga]	bombesin	glioma	[241]
SB3	[^68^Ga]	bombesin	prostate/breast cancer	[243]
DOTA-AE105	[^64^Cu]	uPAR ^g^	breast, prostate, bladder cancer	[248]
NOTA-AE105	[^68^Ga]	uPAR ^g^	breast, prostate, bladder cancer	[249]
DOTA-exendin-4	[^68^Ga]	GLP-1 ^h^	insulinoma	[253]
NOTA-exendin-4	[^68^Ga]	GLP-1 ^h^	insulinoma	[255]
NODAGA-exendin-4	[^68^Ga]	GLP-1 ^h^	Hyperinsulinemic, hypoglycemia	[256]

^a^ Somatostatin subtype 2 receptor, ^b^ neuroendocrine tumours, ^c^ prostate-specific membrane antigen, ^d^ vasoactive intestinal peptide receptor, ^e^ cholecystokinin receptor 2, ^f^ medullary thyroid carcinoma, ^g^ urokinase-type plasminogen activator receptor, ^h^ glucagon-like peptide receptor 1.
